# Do Employees From Less-Healthy Communities Use More Care and Cost More? Seeking to Establish a Business Case for Investment in Community Health

**DOI:** 10.5888/pcd16.180631

**Published:** 2019-07-25

**Authors:** Russell K. McIntire, Martha C. Romney, Greg Alonzo, Jill Hutt, Lauren Bartolome, Greg Wood, Gary Klein, Neil I. Goldfarb

**Affiliations:** 1 Jefferson College of Population Health, Philadelphia, Pennsylvania; 2 Gallagher Benefit Services, Princeton, New Jersey; 3 Greater Philadelphia Business Coalition on Health, Philadelphia, Pennsylvania; 4 CFO Alliance, Wayne, Pennsylvania; 5 Public Health Management Corporation, Philadelphia, Pennsylvania

## Abstract

**Introduction:**

Few studies have examined the impact of community health on employers. We explored whether employed adults and their adult dependents living in less-healthy communities in the greater Philadelphia region used more care and incurred higher costs to employers than employees from healthier communities.

**Methods:**

We used a multi-employer database to identify adult employees and dependents with continuous employment and mapped them to 31 zip code regions. We calculated community health scores at the regional level, by using metrics similar to the Robert Wood Johnson Foundation (RWJF) County Health Rankings but with local data. We used descriptive analyses and multilevel linear modeling to explore relationships between community health and 3 outcome variables: emergency department (ED) use, hospital use, and paid claims. Business leaders reviewed findings and offered insights on preparedness to invest in community health improvement.

**Results:**

Poorer community health was associated with high use of ED services, after controlling for age and sex. After including a summary measure of racial composition at the zip code region level, the relationship between community health and ED use became nonsignificant. No significant relationships between community health and hospitalizations or paid claims were identified. Business leaders expressed interest in further understanding health needs of communities where their employees live.

**Conclusion:**

The health of communities in which adult employees and dependents live was associated with ED use, but similar relationships were not seen for hospitalizations or paid claims. This finding suggests a need for more primary care access. Despite limited quantitative evidence, business leaders expressed interest in guidance on investing in community health improvement.

**Summary**

**What is already known on this topic?**

Peer-reviewed literature discusses economic consequences of poor health and the association between community health, the built environment, and individual health.

**What is added by this report?**

Minimal research has directly examined the effect of poor community health on employees’ medical costs, use of emergency departments, and hospitalizations. We explored whether employed adults and their adult dependents living in less-healthy communities in the greater Philadelphia region used more care and had higher costs than employees from healthier communities.

**What are the implications for public health practice?**

Our findings suggest a need for more primary care access. Business leaders expressed interest for guidance on how to invest in community health improvement.

## Introduction

Peer-reviewed literature discusses the economic consequences of poor health and the association between community health, the built environment, and individual health (1–4). The literature also cites rising costs and lost productivity to employers offering health plan benefits (5,6). Minimal research has examined the effect of poor community health on employees’ medical costs, use of emergency departments (EDs), and hospitalizations. Employers assess the prevalence of illness, service utilization rates, and costs of disease for employees and their dependents but typically do not explore associations between employee health and community health (7,8).

Studies show that 20% of poor health status in impoverished populations is attributable to clinical care, with the remaining 80% attributable to social, economic, and environmental determinants (9,10). Where people live also affects mortality outcomes (10–13). This evidence is an impetus for public and private sectors and the health sector to invest in revitalizing unhealthy communities (14–19).

Although an estimated 80% of employers offer benefits including health management services, the effect on employees’ health status and employer health benefit costs is limited (7,20–22). Employers across business sectors increasingly recognize the impact of community health on employee health, absenteeism and productivity, and the need for a population health approach and investing in community development initiatives (12,15). The Dow Chemical Company, General Electric, Campbell Soup Company, Kaiser Permanente, General Dynamics, Bath Iron Works, and Let’s Move! Active Schools are among the organizations that have initiated projects to address poor community health (7,15,23).

Our study objective was to assess whether employees and their adult dependents living in less healthy communities use more ED and hospital inpatient services and experience higher total claims costs than employees and dependents from healthier communities, among a sample of employees in southeastern Pennsylvania. This research may inform employers about where to invest in communities to improve the built environment and increase access to resources to support healthy living.

## Methods

### Sample

We acquired data from a multi-employer data warehouse, maintained by a benefit consulting organization, containing individual-level data about employees of large employers in southeastern Pennsylvania and their dependents (N = 64,252). The data included demographic characteristics, health care use, and medical cost variables from 2016. We removed data with negative values from the set which were present because of adjustments made from prior time periods. We excluded individuals who did not live in the 5 southeastern Pennsylvania counties (Bucks, Chester, Delaware, Montgomery, Philadelphia) or who only had post office box addresses (n = 61,516). We limited the data set to adults (n = 46,925) with continuous health insurance coverage for the year 2016 (n = 35,845). Finally, we removed 7 people with extreme values of use and cost (n = 35,838).

### Measures

**Individual level**. Variables included in the analyses were those describing demographics (age, sex [male or female], and relative status [employee, spouse, or adult child]), medical claims costs, number of inpatient hospitalizations, and number of ED visits. We treated sex and relative status as categorical variables. All other variables were treated as continuous.

**Zip code region level.** Using Environmental Systems Research Institute’s ArcGIS version 10.3 mapping software (Esri), the zip codes in southeastern Pennsylvania were aggregated to create 31 zip code regions (zip regions), which have been used previously by the City of Philadelphia and the Public Health Management Corporation (PHMC) to summarize local health data (24). We developed a summary community health variable (Health Index) based on methods used by the Robert Wood Johnson Foundation (RWJF) County Health Rankings, but using local measures available at the zip code level aggregated to zip regions based on proximity. We replicated the structure of the County Health Rankings health outcomes and health factor domains, which included health outcomes, health behaviors, clinical care, social and economic environments, and physical environment. Each domain was composed of 5 to 9 component measures. We found local data at the zip code level, such as the PHMC Household Health Survey (25) and US Census data (26) to cover 66.6% of the component measures included in the RWJF County Health Rankings methodology. We then weighted the measures and domains per RWJF Health Rankings methods (27).

To link individual- and zip region–level data, we used ArcGIS to geocode adult employees and dependents by address to identify the zip region in which they resided in 2016. We used a consensus-based approach among all stakeholders and researchers involved to identify the statistical analysis plan, including treatment of variables and the outliers among the outcome variables. Based on analyses of the existing data and knowledge of the outcome variables, we eliminated cases with the following characteristics: >$800,000 in medical claims (n = 1), >15 inpatient hospitalizations (n = 1), and >20 ED visits (n = 5). The final sample was 35,838. 

We conducted a focus group to present data findings to financial and human resource business executives convened by The CFO Alliance, representing employers from various sectors of the economy and of differing sizes. The Thomas Jefferson University institutional review board granted approval for the study.

### Statistical analysis

We conducted descriptive statistical analyses using SPSS version 23 (IBM Corporation) and statistical modeling using SAS version 9.4 (SAS Institute, Inc). We used descriptive statistics to summarize each individual- and zip region–level variable, including counts, proportions, means, and standard deviations. We used simple linear regression to explore relationships between demographics, the Health Index variable, and each dependent variable. Independent variables that showed a significant bivariate relationship (at *P* < .10) with dependent variables were retained for multilevel analysis. We tested bivariate relationships between the 5 domains that comprised the Health Index and each dependent variable. We used multilevel linear modeling to identify the direct effects of individual-level demographics (age and sex) and the group-level factor of community health (Health Index) on the dependent variables: medical claims costs, number of inpatient hospitalizations, and number of ED visits, respectively (fixed effects). We also conducted multilevel models to see if each domain individually predicted each of the dependent variables. Because the individual-level data set did not include a variable for race, we controlled for the proportion of white residents at the zip region level using American Communities Survey 5-year estimates (2011–2015).

We considered using zero-inflated models because of the high proportion of zero values in the dependent variables, especially for inpatient hospitalizations. Because zero-inflated models cannot distinguish between variance at the individual and group levels, we used multilevel linear modeling. We calculated an intraclass correlation coefficient (ICC) to estimate the amount of variance in outcomes that were accounted for by zip regions. We assessed whether clusters of families should be considered in the models by randomly selecting an individual per household, conducting the models again, and checking model coefficients and ICCs. Results were similar, so we ignored household clustering. A random effect for intercepts was included for zip regions. The sample size per zip region ranged from 79 to 3,984, with a mean of 1,156. Significance was set at *P* < .05 for multilevel models.

## Results

Most of the sample of employees and adult dependents was female (55.6%) and of working age (18–39, 40.5%; 40–59, 44.1%) (Table 1). The age range was 18 to 94 years. The sample was unequally geographically distributed, with most living in Delaware (29.2%) and Bucks (24.6%) Counties. The mean medical claims cost among the sample was $4,803.41. In 2016, the mean number of ED visits per person was 0.31, and the mean number of inpatient hospitalizations per person was 0.07.

**Table 1:** Demographic and Outcome Variables of Adult Employees and Dependents in Southeastern Pennsylvania, 2016

Variable	Value^a^
**Female sex (N= 35,838)**	19,925 (55.6)
**Age, y (N= 35,838)**	
18–39	14,495 (40.5)
40–59	15,820 (44.1)
≥60	5,523 (15.4)
**County (N= 35,838)**	
Bucks	8,800 (24.6)
Chester	6,993 (19.5)
Delaware	10,461 (29.2)
Montgomery	6,791 (19.0)
Philadelphia	2,793 (7.8)
**Relative status (N = 35,838)**	
Employee	19,606 (54.7)
Spouse	10,405 (29.0)
Child (>18 y)	5,827 (16.3)
**Medical claims cost, mean (standard deviation), $ (n = 35,833)^b^**	4,803.41 (18,105.18)
**Number of emergency department visits (n = 35,837)^b^**	0.31 (0.93)
**Number of inpatient hospitalizations (n = 35,837)^b^**	0.07 (0.37)

a Values are mean number (%) unless otherwise indicated.

b Negative values removed per outcome analysis.

**Figure 1:**
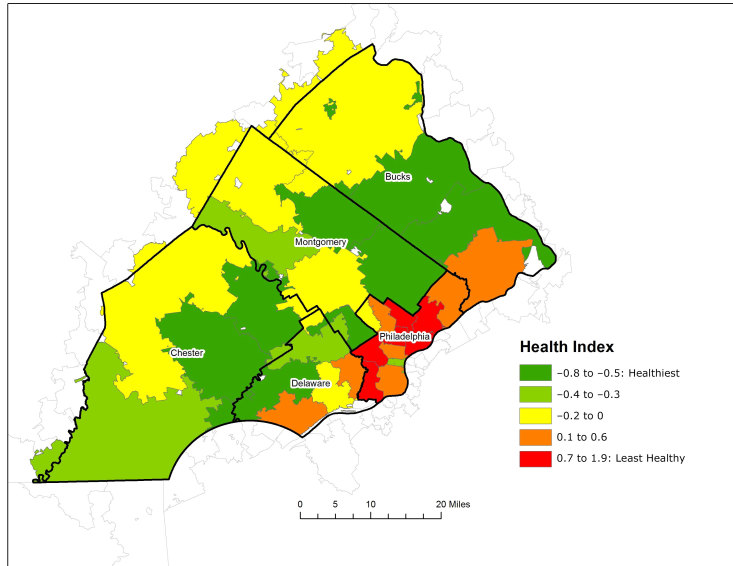
The Health Index by zip region (n = 31), southeastern Pennsylvania, 2016. Data sources: PHMC Household Health Survey and American Communities Survey, 5-Year Estimates 2011–2015.

We identified community health disparities among the 31 zip regions in southeastern Pennsylvania (Figure 1). Overall, Philadelphia County zip regions had the highest Health Index scores, denoting the poorest health, followed by zip regions in eastern Delaware County and southern Bucks County.

**Figure 2:**
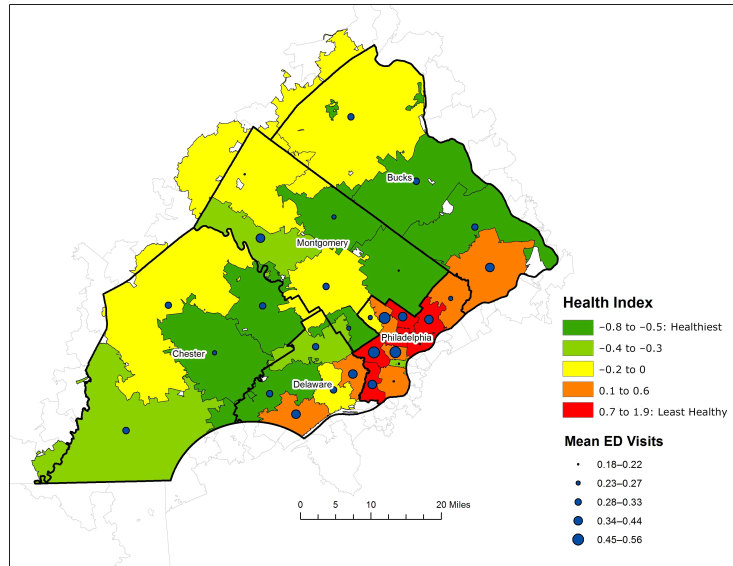
Mean number of emergency department visits by the Health Index among adults that live in zip regions in southeastern Pennsylvania. Abbreviation: ED, emergency department. Data sources: PHMC Household Health Survey and American Communities Survey, 5-Year Estimates 2011–2015.

We found a clear positive relationship between the Health Index and mean ED visits (Table 2). This relationship was confirmed through simple linear regression (data not shown), which identified a significant relationship between the Health Index of the zip region in which individuals lived and mean ED visits. Mean ED visits by zip region increased by 0.064 for each 1-unit increase in Health Index (denoting poorer community health) (β = 0.064, *P* = .009). Figure 2 shows the relationship between Health Index and mean ED visits by zip region. Healthier zip regions (green and yellow) had lower mean ED visits (smaller dots), whereas zip regions with poorer health (red and orange) have higher mean ED visits (larger dots).

**Table 2:** Summary Statistics of Mean Emergency Department Visits, Inpatient Hospitalizations, and Total Medical Claims Costs Among Employees and Adult Dependents (N = 35,838), by Zip Region Quartiles and Health Index, Southeastern Pennsylvania, 2016

Zip Code Region	Mean No. Emergency Department Visits Per Adult	Mean No. Hospitalizations Per Adult	Mean Total Paid Claims Per Adult, $
1st Quartile (healthiest zip regions)	0.273	0.071	4,645
2nd Quartile	0.284	0.064	4,144
3rd Quartile	0.339	0.071	4,438
4th Quartile (least healthy zip regions)	0.443	0.060	4,008

No clear descriptive or bivariate statistical relationship for mean hospitalizations or mean total claims by health of zip region was found, including overall Health Index or any of the health domains. Thus, we explored relationships between community health and the ED visits outcome only using multilevel modeling.

We used multilevel models to explore the relationship between the Health Index of zip regions and frequency of ED visits after controlling for individual-level demographics (age and sex) (Table 3). Most of the variation in ED visits was at the individual level (ICC = 0.002). Model 1 identified an inverse relationship between age and ED frequency; as age decreased, the frequency of ED visits increased (β = −0.002, SE = 0.0003). Being female was associated with increased frequency of ED visits (β = 0.055, SE = 0.010). After controlling for individual-level demographics, we found that a 1-unit increase in the Health Index (representing poorer community health) was associated with increased frequency of ED visits by 0.077 (β = 0.077, SE = 0.019). However, after including a summary measure of the proportion of white residents by zip region in the multilevel model (Table 3), the significant relationship between Health Index and ED visits became nonsignificant.

**Table 3:** Multilevel Linear Regression Model Predicting Emergency Department Visits Among Employees and Adult Dependents (N = 35,838), Southeastern Pennsylvania, 2016

Independent Variable	Model 1		Model 2	
	β	Standard Error	β	Standard Error
**Individual level**				
Age	−0.002	0.0003^a^	−0.002	0.0003^a^
Female (Reference group: male)	0.055	0.010^a^	0.055	0.010^a^
**Zip region level**				
Health index	0.077	0.019^a^	0.009	0.031
% White	—	—	−0.002	0.001^b^

Abbreviation: — , not applicable.

a *P* < .001

b *P* < .01

Each of the Health Index domains (health outcomes, health behaviors, clinical care, social and economic environment, and physical environment) significantly predicted ED visits except clinical care. The significant relationships also became nonsignificant after controlling for the proportion of white residents (data not shown).

Executives in the focus group were not surprised by an association between ED use and community health but were surprised that a similar relationship was not found for the other outcome variables (hospital use and total paid claims). They expressed a high level of interest in understanding the health needs of the communities in which their employees live and wanted to see developing evidence about how to identify need, where to invest, and how to measure the return on investment. Most executives agreed that there is opportunity to use community and population health improvement to drive increased corporate philanthropy locally. 

To inform future studies, employers also expressed an interest in statistical analyses of these data relative to family income, opioid use, end-of-life claims, and other disease states, such as diabetes and cancer. Several of the financial and human resource executives expressed relief that the hypothesis regarding total claims cost was not proven. The prevailing view of most employers was that a positive finding would be more likely to lead to community health investment than community abandonment. Employers expressed that a strategic plan and guidance on how to implement change in their companies and communities, leveraging models from other communities and using existing financial and educational resources and government incentives for support, would be useful.

## Discussion

Although many studies have investigated the relationships between community health and individual health, this is one of the first to explore the outcomes of employee use of services and direct medical costs. We found that employees living in areas with poorer community health (and the domains of community health including poorer health outcomes, health behaviors, social and economic environment, and physical environment) had higher ED utilization. No relationships were found between community health and hospital utilization or total medical cost (paid claims). Analyses suggest that other intervening variables such as racial composition of a community may help to explain the community health–ED utilization relationship. Nonetheless, for employers, community health serves as an important descriptive marker for ED utilization.

Public Health 3.0 challenges the public health community to develop partnerships with multiple sectors, including the business community, and to plan and implement public health improvement initiatives (28). The study provides information that can drive collaborative efforts between employers and other public health stakeholders. Given that ED use is a direct and indirect cost (lost productivity) concern for many employers (5,29), the findings may help to focus future efforts on reduction of ED use by employees and dependents in less-healthy communities. These efforts can include reducing barriers to primary care services through investing in worksite or community-based primary care clinics, expanding coverage for retail clinics and urgent care centers, and offering telemedicine services. For example, General Electric invested in patient-centered medical homes and technology, resulting in increased access to preventive/ambulatory health services, fewer ED visits, and increased worker productivity (7,21). Employers should work with researchers to develop and provide employee education about the importance of primary care and understanding appropriate use of the ED; such programs are implemented by the Massachusetts Employer-Led Coalition to Reduce Health Care Costs (30) and the Midwest Health Initiative (31), among others.

One concern expressed by team members in designing this study, and by reviewers of the original grant proposal, was that the potential finding that workers from less-healthy communities cost more could lead some employers to relocate their businesses or introduce biases into their employee recruitment and hiring processes. However, many employers are physically, culturally, or otherwise tied to their communities, and expressed a commitment to fostering healthy communities. The challenge is not to prove the impact of community health on employers, but rather to demonstrate which actions and investments are most likely to have a measurable positive impact on health to yield a return on investment.

This study has several limitations. First, demographic characteristics of the individuals in the study, beyond age, sex, and relative status, were not available for analysis. This is a clear limitation as individual-level demographics (such as race/ethnicity or income) could have confounded our results. Where possible, we used aggregate, community-level demographics to remove some of the potential for confounding, but we do not know if, or to what extent, individual employees were exposed to community-level risk factors that make up the domains which composed the Health Index. We also did not have information on co-insurance (eg, Medicare) or the type of benefit plan design (eg, health maintenance organization, preferred provider organization, consumer-driven health plan, high-deductible health plan), which would determine cost-sharing among the employee, employer, and payer. Second, we focused our analysis on employees and adult dependents who were covered during a 12-month period. This approach allowed us to summarize results of employees that were regularly and continuously employed and covered by insurance, but it removed employees whose employment was not continuous, possibly some who may have stopped working due to poor health. Additionally, we did not remove pregnant women or those with acute or chronic conditions, as part of the increased costs of the employees’ care that was incurred by employers. Third, we used modified RWJF County Health Rankings methods to create the Health Index variable. For two-thirds of the measures we found appropriate local data at the zip code level, but one-third of the measures were not identified. Thus, the Health Index domains may not mirror the health outcome and health factor domains generated by the RWJF County Health Rankings methods. Fourth, although we examined health across 31 zip code clusters from 5 counties in southeastern Pennsylvania, the clusters with the poorest health were confined largely to Philadelphia County, which also had the smallest number of cases. Therefore, the study may have had limited power to examine community health as an independent risk factor for utilization, and results may not be generalizable to broader geographies and less-urban regions. Fifth, our outcome variables were highly skewed because of zero values. We used means to describe our outcome variables (as opposed to medians) because the major goal of the study was analytic: to identify the relative relationships between community health and our outcome variables. Sixth, the analysis was based on cross-sectional data from 1 year, so results are limited to associations only and causation between variables cannot be inferred.

Despite these limitations, results suggest that community health was correlated with employee health among our sample of employees in southeastern Pennsylvania, at least with regard to ED utilization rates. Future research should further explore the mechanisms behind these relationships and develop and test strategies for business investment in building healthier communities. Additionally, future studies exploring these relationships should consider the influence of benefit plan design, which may offer insight into relationships of employee out-of-pocket costs relative to ED use and hospitalizations.
